# Can Clinical, Psychological, and Cognitive Patient-Reported Outcome Measures (PROMs) Help to Discriminate Women with Fibromyalgia from Those with Other Localized/Regional Pain Conditions? A Diagnostic Accuracy Study

**DOI:** 10.3390/medicina61020359

**Published:** 2025-02-19

**Authors:** Margarita Cigarán-Mendez, Ángela Tejera-Alonso, Cristina Gómez-Calero, César Fernández-de-las-Peñas, Mónica López-Redondo, Juan A. Valera-Calero, Francisco G. Fernández-Palacios, Juan C. Pacho-Hernández

**Affiliations:** 1 Department of Psychology, Universidad Rey Juan Carlos, 28922 Alcorcón, Spain; margarita.cigaran@urjc.es (M.C.-M.); angela.tejera@urjc.es (Á.T.-A.); gines.fernandez@urjc.es (F.G.F.-P.); juancarlos.pacho@urjc.es (J.C.P.-H.); 2 Escuela Internacional de Doctorado, Universidad Rey Juan Carlos, 28922 Alcorcón, Spain; 3 Department Physical Therapy, Occupational Therapy, Rehabilitation, and Physical Medicine, Universidad Rey Juan Carlos, 28922 Alcorcón, Spain; cristina.gomez@urjc.es; 4 Faculty of Health Sciences, Universidad Francisco de Vitoria, 28223 Madrid, Spain; monica.lopezredondo@ufv.es; 5 Department of Radiology, Rehabilitation and Physiotherapy, Faculty of Nursery, Physiotherapy and Podiatry, Complutense University of Madrid, 28040 Madrid, Spain; juavaler@ucm.es; 6 Grupo InPhysio, Instituto de Investigación Sanitaria del Hospital Clínico San Carlos (IdISSC), 28040 Madrid, Spain

**Keywords:** fibromyalgia, diagnostic accuracy, pain, disability, hypervigilance, anxiety, depression, sleep

## Abstract

*Background and Objectives:* The heterogeneous clinical manifestations of fibromyalgia syndrome have led to the revision of diagnostic criteria in the last decade. The aim of this study was to determine the capability of clinical, psychological, and cognitive patient-related outcome measures (PROMs) to differentiate women with fibromyalgia syndrome (FMS) from women with localized or regional pain conditions. *Materials and Methods:* A diagnostic accuracy study was conducted. Clinical (pain intensity—NPRS; related disability—FIQ), psychological (anxiety/depressive levels—HADS-A/HADS-D), and cognitive (sleep quality—PSQI; pain hypervigilance—PVAQ-9) PROMs were collected in 129 women with FMS and 65 women with localized/regional chronic pain conditions. The area under the receiver operating characteristic (ROC) curve, cut-off point, sensitivity/specificity values, and positive and negative likelihood (LR) ratios of each variable were calculated. *Results:* Women with FMS showed higher levels of pain, related disability, and anxiety/depressive levels, worse sleep quality, and higher levels of hypervigilance (all, *p* < 0.001) than women without FMS. All PROMs showed excellent discriminatory power and good sensitivity (pain intensity: ROC 0.987, sensitivity 91.5%; related disability: ROC 0.980, sensitivity 93.8%; HADS-A: ROC 0.901, sensitivity 81.4%; HADS-D: ROC 0.906, sensitivity 85.3%; PSQI: ROC 0.909, sensitivity 79.1%; PVAQ-9: ROC 0.798, sensitivity 80.6%). Specificity was extremely small for all variables (<18%) except for pain hypervigilance (specificity: 34%). *Conclusions:* Women with FMS exhibited worse clinical, psychological, and cognitive variables than women with localized/regional chronic pain. Although all PROMs had good discriminatory power, related disability and pain hypervigilance were those showing the best models. These PROMs could be combined with the American College of Rheumatology (ACR) diagnostic criteria to better discriminate between women with and without FMS. Studies investigating the relevance of combining these PROMs with the ACR diagnostic criteria in clinical settings are needed.

## 1. Introduction

Fibromyalgia syndrome (FMS) is a chronic pain condition characterized by a plethora of symptoms such as fatigue, diffuse widespread pain, muscle stiffness, sleep disturbances, psychological disorders, and cognitive dysfunctions. The heterogeneous clinical manifestations of FMS have led to changes in the original diagnostic criteria published in 1990 by the American College of Rheumatism (ACR) [1]. In 2010, the ACR proposed an update version of the diagnostic criteria based exclusively on the use of two self-reported scales assessing the presence of widespread symptoms [2]. In 2016, an updated version of the ACR diagnostic criteria was published [3]. Salaffi et al. reported good sensitivity and specificity and proper classification of patients with FMS (78%, 90.5%, and 83.6%, respectively) when applying the diagnostic criteria ACR of 2016 [4]. However, these updated diagnostic criteria are also questioned. In fact, Cassisi et al. have recently identified inaccuracy in FMS diagnosis using the ACR 2016 diagnostic criteria, highlighting the possibility of a high risk of classifying individuals with chronic pain but without FMS as having FMS [5]. These authors observed the presence of clinical findings compatible with FMS in individuals who do not meet the ACR criteria [5].

In fact, some authors have attempted to identify symptoms to be specifically associated with FMS. Bennett et al. identified that persistent deep pain, poor balance, environmental sensitivity, tenderness to touch, and pain after exercise were the potential symptoms which best differentiated between patients with FMS from individuals with other pain conditions [6]. Jones et al. also identified that persistent deep aching pain was a cardinal symptom able to differentiate between patients with FMS and other localized chronic pain conditions [7]. Since FMS is a complex pain condition where clinical, psychological, and cognitive variables are associated [8,9], it is possible that the use of these variables could also help to differentiate this condition from other chronic pain conditions.

Patient-reported outcome measures (PROMs) are defined as self-reported questionnaires used for evaluating different aspects of a particular condition. These PROMs can be generic (when evaluating general aspects of mental/physical health) or disease-specific (when assessing symptoms relevant to a particular condition) [10]. Churruca et al. identified up to 315 generic and disease-specific PROMs used in health sciences [11]. The Outcome Measures in Rheumatology (OMERACT) initiative was developed with the aim of creating a consensus in the use of PROMS in FMS in clinical trials [12]. The OMERACT identified that pain, fatigue, global health status, function, and sleep disturbances are domains which should be assessed in FMS [12]. A recent review has identified the use of 126 different PROMs for evaluating 50 domains in individuals with FMS, with pain, fatigue, and depressive/anxiety levels being the most frequently assessed domains in >95% of clinical trials [13]. This review also identified that the Fibromyalgia Impact Questionnaire (FIQ) was the disease-specific PROM most frequently used (82%), and the Numerical Pain Rate Scale (NPRS) was the most frequently generic PROM evaluated (51.4%) in clinical trials of FMS [13].

The U.S. National Institutes of Health-funded Patient-Reported Outcome Measurement Information System^®^ (PROMIS^®^) introduced emotional health as a relevant domain to also be assessed in chronic pain conditions such as FMS [14]. Thus, Döhmen et al. observed that depressive symptoms was a domain assessed in up to 98.1% of clinical trials including subjects with FMS, whereas anxiety level was assessed in 95.2% of clinical trials including subjects with FMS [13]; however, heterogeneous generic PROMs, e.g., the Hospital Anxiety and Depression Scale (HADS) or the State Trait Anxiety Inventory (STAI), were used for that purpose.

This study aimed to determine the capability of different generic PROMs covering the psychological (anxiety and depressive levels), cognitive-related (sleep quality or pain hypervigilance), and clinical (pain intensity) domains and one disease-specific PROM covering related disability (FIQ) to differentiate women with FMS from those with other localized/regional chronic pain conditions by analyzing the area under the receiver operating characteristic (ROC) curve, cut-off point, sensitivity/specificity scores, and positive/negative likelihood (LR) ratios for each PROM. A secondary objective was to determine the association between these PROMs to identify potential predictors of those variables showing the best ability according to the prediction models.

## 2. Materials and Methods

### 2.1. Participants

A group of women diagnosed with FMS by their rheumatologists according to the 2016 ACR diagnostic criteria [3] was recruited from various Fibromyalgia Associations in Madrid, Spain. Additionally, a group of women experiencing localized (e.g., knee or shoulder pain) or regional (e.g., lower back pain) chronic pain without a confirmed FMS diagnosis (as verified by their doctor) were recruited via local social media platforms. Participants were excluded if they had (1) a history of whiplash injury; (2) prior surgeries; (3) any underlying medical condition; (4) neuropathic pain such as radiculopathy; or (5) a current psychiatric diagnosis according to the DSM-V (e.g., schizophrenia, major or mild neurocognitive disorders), or (6) were using medications (e.g., antipsychotics, anticonvulsants, anticholinergics) that could influence cognitive function. The study design received ethical approval from the Local Ethics Committee at the Universidad Rey Juan Carlos (URJC code 2508202218222), and all participants signed a written informed consent form before taking part.

### 2.2. Clinical-Related Patient-Reported Outcome Measures

The generic patient-reported outcome measure (PROM) for clinical evaluation was pain intensity. Participants assessed their average pain intensity at rest, their worst pain intensity at rest, and the pain they experienced during daily activities using separate 11-point numerical rating scales (NPRS—0: no pain; 10: worst possible pain) [15]. The mean of these three values was calculated for the main analysis [16].

The specific disease-related clinical PROM focused on disability and functioning. The Spanish version of the Fibromyalgia Impact Questionnaire (FIQ), which ranges from 0 to 100 points, was employed to evaluate functional status and related disability [17]. The FIQ comprises 10 items that assess physical function, days of feeling unwell, missed work, social interactions, fatigue, morning stiffness, tightness, anxiety, and depression. Higher scores indicate a greater negative impact of FMS [18].

### 2.3. Psychological and Cognitive Patient-Reported Outcome Measures

Anxiety and depression levels were measured using the Spanish version of the Hospital Anxiety and Depression Scale (HADS) [19]. This PROM consists of two subscales (HADS-A for anxiety and HADS-D for depression), each comprising 7 items scored from 0 to 3 points. The scale’s psychometric properties have demonstrated reliability in detecting symptoms of anxiety and depression in the general population [20].

Sleep quality was assessed using the Spanish version of the Pittsburgh Sleep Quality Index (PSQI) [21], which generates an overall score (ranging from 0 to 21) based on responses to 19 questions covering various aspects of sleep [22].

To evaluate pain hypervigilance, the Spanish version of the 9-item Pain Vigilance and Awareness Questionnaire (PVAQ-9) was used. This tool provides a score between 0 and 45 points and evaluates tendencies to observe, monitor, and focus on pain [23].

### 2.4. Statistical Analysis

All data processing and statistical analyses were performed using SPSS version 29.1.1 (Armonk, NY, USA) for Mac OS. Continuous variable distributions were assessed using histograms and the Shapiro–Wilk test. Differences in clinical, psychological, and cognitive PROMs between groups (women with and without FMS) were examined using independent Student’s *t*-tests. Due to the inclusion of multiple comparisons, a two-tailed *p*-value of less than 0.008 (Bonferroni correction, *p* = 0.05/6) was considered statistically significant.

The first step involved evaluating the diagnostic accuracy of these PROMs in differentiating between women with and without FMS. This step was achieved by analyzing the area under the receiver operating characteristic (ROC) curve, where ROC values of 0.7 or higher indicate acceptable discriminatory ability [24]. The optimal cutoff points for each variable were determined by using the Youden index [25]. Sensitivity, specificity, and positive and negative likelihood ratios (LRs) were also calculated. Validity was considered acceptable when sensitivity was at least 70% and specificity exceeded 50% [26].

In the second step, multiple linear regression was applied to identify factors contributing to variance in PROMs with the strongest diagnostic performance. Correlations between all PROMs (independent and dependent variables) were calculated using Pearson correlation coefficients (r). Those PROMs significantly correlated with the dependent variables (pain intensity measured by NPRS and disability measured by FIQ) were included in stepwise regression analyses. Correlation analyses were also used to identify multicollinearity and shared variance, defined as r > 0.8. Variables showing multicollinearity were excluded. The threshold for significance in regression equations was also set at *p* < 0.008. Adjusted R^2^ changes after each step of the regression model were reported to explain the contribution of additional variables [27]. All correlation and regression analyses were conducted separately for the groups of women with and without FMS.

## 3. Results

### 3.1. Participants

From an initial sample of 150 women with FMS screened according to the eligibility criteria, a total of 1291 women with FMS (age: 54.7, SD: 9.4 years) were finally included. Thus, from an initial sample of 100 women with chronic pain but without FMS screened, a total of 65 women with localized/regional chronic pain (mean age: 59.7; SD: 9.5 years) fulfilled all the inclusion criteria. The localized/regional pain conditions within the non-FMS group were chronic neck pain (*n* = 21, 32.3%), knee pain (*n* = 17, 26.2%), chronic low back pain (*n* = 12, 18.5%), shoulder pain (*n* = 7, 10.8%), headache (*n* = 4, 6.1%), and temporomandibular pain (*n* = 4, 6.1%).

Table 1 summarizes the comparison of clinical, psychological, and cognitive-related variables between women with and without FMS. Women with FMS showed higher levels of pain, longer pain duration, higher disability and anxiety and depressive levels, worse sleep quality, and higher levels of pain hypervigilance (all, *p* < 0.001) than those women with chronic pain but without FMS.

### 3.2. Diagnostic Accuracy Analysis

Table 2 details the diagnostic accuracy information of PROMs in distinguishing women with and without FMS using ROC curve analysis. Considering that ROC values ≥0.7 are considered acceptable for diagnostic purposes, the results indicate that all PROMs assessed possess strong discriminatory power for differentiating women with and without FMS. The most notable PROMs were pain intensity (NPRS) and related disability (FIQ), which exhibited particularly high ROC values (0.987 and 0.980, respectively). These ROC values suggest that both are effective at identifying women with FMS. Pain intensity showed a high sensitivity (91.5%), meaning it correctly identifies most women with FMS, but a low specificity (3.6%), indicating that it was less effective at ruling out women without FMS but with localized/regional chronic pain. Similarly, disability (as assessed with a disease-specific PROM such as FIQ) showed excellent sensitivity (93.8%) but also low specificity (5.4%), reflecting the same pattern of strong identification of women with FMS but limited exclusion of those without FMS and other chronic pain conditions.

Psychological variables, such as HADS-A, HADS-D, and PQSI, also demonstrated high discriminatory power, with ROC values above 0.9. The HADS-D had a ROC of 0.906, and a sensitivity of 85.3%, indicating good accuracy in identifying women with FMS, but again, its specificity was relatively low (18%). The HADS-A performed similarly, with an ROC of 0.901, a sensitivity of 81.4%, and a specificity of 17%. The PSQI had a ROC of 0.906, a sensitivity of 79.1%, and a specificity of 11.7%. Finally, pain hypervigilance, while still showing acceptable performance, had the lowest ROC value (0.798) compared to the other PROMs. Nevertheless, this variable had moderate sensitivity (80.6%) and somewhat better specificity (34.2%) than the others. However, its Youden Index (0.464) suggested that it was less balanced overall (Table 2).

**Figure 1:**
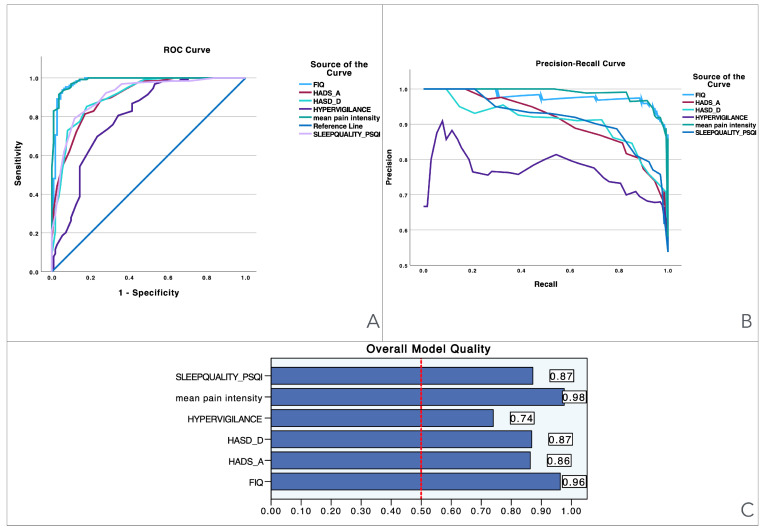
Visualization of model quality by using ROC curves and precision–recall curves for pain intensity (NPRS), related disability (FIQ), anxiety levels (HADS-A), depressive levels (HADS-D), sleep quality (PQSI), and pain hypervigilance (PVAQ-9). The ROC curve (**A**) shows the trade-off between sensitivity and specificity for each variable, while the precision–recall curve (**B**) further details the performance of these variables. The bar charts (**C**) quantify the overall model quality for each variable.

To provide visual information, Figure 1 illustrates the performance of various predictors across three evaluation metrics: the ROC curve, precision–recall curve, and overall model quality. The Precision–recall curves plot precision (the ratio of true positives to all predicted positives) against recall (the ratio of true positives to all actual positives) across different threshold settings. They are particularly useful for evaluating models on imbalanced datasets, where one class is much rarer than the other. Unlike ROC curves, which may be overly optimistic in such cases, precision–recall curves focus on the performance of the model with respect to the positive class, offering a clearer view of its ability to correctly identify positive instances while minimizing false positives. The area under the precision–recall curve provides a summary measure of the model’s performance, highlighting its effectiveness in handling the positive class. Panel A presents the ROC curves, showing the trade-off between sensitivity and specificity for each predictor, with pain intensity achieving the best performance (light blue line), followed by related disability and sleep quality. Panel B shows the precision–recall curve, highlighting the precision against recall across different thresholds, where pain intensity again demonstrates superior performance, maintaining high precision and recall when compared to the other variables. Finally, Panel C displays the overall model quality, where pain intensity has the highest score (0.98), followed by related disability (0.96) and sleep quality (0.87), while hypervigilance performs the lowest (0.74). These results suggest that pain intensity and related disability are the most reliable predictors, outperforming measures like pain hypervigilance and mood-related scales.

### 3.3. Bivariate Correlation Analysis

The bivariate correlation analyses for women with FMS are summarized within Table 3. Pain intensity (NPRS) was positively associated with all PROMs (all, *p* < 0.001) (disability (r = 0.456), anxiety levels (r = 0.404), depressive levels (r = 0.339), pain hypervigilance (r = 0.420), and sleep quality (r = 0.378)): the higher the intensity of pain, the higher the related disability, the higher the anxiety/depressive levels, the higher the pain hypervigilance levels, and the poorer the sleep quality. Similarly, disability was also positively associated with the remaining PROMs (all, *p* < 0.001) (anxiety levels (r = 0.500), depressive levels (r = 0.512), pain hypervigilance (r = 0.276), and sleep quality (r = 0.482): the higher the related disability, the higher the anxiety/depressive levels, the higher the pain hypervigilance levels and the poorer the sleep quality within women with FMS. No multicollinearity (defined as r > 0.8) between PROMs was identified; therefore, all PROMs were included in the multiple regression analysis.

The bivariate correlation analyses for women with localized/regional chronic pain are shown in Table 4. Pain intensity (NPRS) was positively associated with all PROMs (all, *p* < 0.001) (disability (r = 0.564), anxiety (r = 0.457) and depressive (r = 0.356) levels, pain hypervigilance (r = 0.271), and sleep quality (r = 0.390)): the higher the intensity of pain, the higher the related disability, the higher the anxiety/depressive levels, the higher pain hypervigilance levels, and the poorer the sleep quality. Similarly, disability was also positively associated with the remaining PROMs (all, *p* < 0.001) (anxiety (r = 0.745) and depressive (r = 0.580) levels, pain hypervigilance (r = 0.410), and sleep quality (r = 0.441): the higher the related disability, the higher anxiety/depressive levels, the higher the pain hypervigilance levels, and the poorer the sleep quality within women with FMS. No multicollinearity (defined as r > 0.8) between PROMs was identified; therefore, all PROMs were included in the multiple regression analysis.

### 3.4. Multiple Regression Analysis

The hierarchical regression analysis to determine the explained variance of pain intensity and disability in women with FMS is shown in Table 5. Stepwise regression analyses revealed that disability (contributing 20.2%) and pain hypervigilance (an additional 8.7%) contributed significantly to pain intensity, and, when combined, explained 28.9% of its variance (r2 adjusted: 0.289). Thus, depressive levels (contributing 25.6%), pain intensity (an additional 8.6%), and sleep quality (an additional 3.8%) contributed significantly to related disability, and, when combined, explained 38% of its variance (r2 adj: 0.380) in women with FMS.

The hierarchical regression analysis to determine the explained variance of pain intensity and disability in women with localized/regional pain is shown in Table 6. Stepwise regression analyses revealed that disability was the only PROM significantly contributing to pain intensity, explaining 30.7% of its variance (r2 adjusted: 0.307). Thus, anxiety levels (contributing 54.9%), pain intensity (an additional 5.7%), and pain hypervigilance (an additional 2.1%) contributed significantly to related disability, and, when combined, explained 62.7% of its variance (r2 adj: 0.627) in women without FMS.

## 4. Discussion

The current study found that women with FMS exhibit worse clinical (higher intensity of pain and more related disability), psychological (higher anxiety and depressive levels), and cognitive-related (poor sleep quality, higher pain hypervigilance level) variables than women with other chronic pain conditions. In addition, all the PROMs used in this study showed ROC values ≥ 0.9, indicating discriminatory power to differentiate women with FMS from women with other chronic pain conditions but without FMS; however, the specificity values were extremely low, indicating that while these PROMs can help to discriminate women with FMS, they cannot reliably confirm the absence of FMS in women with other chronic pain conditions. Finally, related disability, assessed with a specific-disease PROM such as the FIQ, and pain hypervigilance, as assessed with a generic PROM such as PVAQ-9, were those variables showing the best predictive models.

### 4.1. Clinical PROMs in FMS: Pain Intensity and Related Disability

We found that women with FMS exhibit higher pain intensity and more disability than women with other chronic pain conditions but without FMS. Our results agree with Pontes-Silva et al., who also observed that FMS patients (2016 ACR criteria) also reported higher levels of pain and fatigue, worse function, and worse symptom severity when compared with patients with other musculoskeletal chronic pain conditions [28]. It should be noted that the pain intensity of our group of women with FMS was over twice the pain intensity of our cohort of women with chronic pain conditions but not FMS. Pontes-Silva et al. reported a similar pain intensity value (7.5 for pain with movement) to our sample [28]. The current literature shows that individuals with FMS usually exhibit higher levels of pain [6,7], but it is possible that our sample represents a spectrum of FMS women with high levels of pain and related disability. Pain intensity was evaluated with NPRS, a generic PROM commonly used in clinical trials [13]. In fact, this PROM showed the highest ROC value (0.987) and a high sensitivity score (91.5%), meaning that it has good discriminatory power to differentiate between women with and without FMS. However, its specificity value was extremely low (3.6%), indicating that high pain intensity can help to identify the presence of FMS, but the presence of a higher level of pain cannot reliably rule out women without FMS but with other localized/regional chronic pain conditions.

Similarly, related disability as assessed with the FIQ also had good discriminatory power to differentiate between women with and without FMS, showing an ROC value of 0.980 and excellent sensitivity (93.8%). However, its specificity was extremely low (5.4%), indicating that while the FIQ can help to identify the presence of disability related to FMS, it itself cannot reliably exclude the presence of disability in women with localized/regional pain but without FMS. In our study, we used the FIQ as a disease-specific PROM, since the FIQ is the most frequently used (82%) in clinical trials of FMS [13]. The revised FIQ (FIQ-R) was created in 2009 and included other domains such as memory, tenderness, balance, and environmental sensitivity [29]. In fact, the Spanish version of the FIQ-R has shown high internal consistency, good test–retest reliability, and significant correlation with the FIQ [30]. Although the FIQ-R covers more domains than the original FIQ, today, the FIQ is the PROM used most for evaluating related disability in FMS [13].

The bivariate analyses revealed that these PROMs, NPRS and FIQ, were moderately associated in both groups, with slightly higher correlation within the non-FMS group. In fact, the regression analysis revealed that disability was the only significant predictive factor for pain intensity in the group of women with localized/regional pain conditions (see Table 6), whereas disability and pain hypervigilance were predictors for pain intensity in women with FMS (see Table 5). These differences could be related to the fact that FMS is a more complex chronic pain condition than those localized/regional pain conditions experienced by the non-FMS group and a more direct association between pain and disability is observed in the latter.

### 4.2. Psychological and Cognitive PROMs in FMS

Our results also revealed that women with FMS exhibited higher anxiety and depressive levels, poor sleep quality, and higher level of pain hypervigilance when compared to women with other localized/regional chronic pain conditions but without FMS. The presence of anxiety/depressive symptoms [31] and poor sleep quality [32] in women with FMS is not new and it is clearly supported in the literature.

In fact, anxiety/depressive levels are evaluated in up to 95% of clinical trials including patients with FMS [13]; however, these symptoms are evaluated with heterogeneous PROMs. The results of this study found that these PROMs could help to better differentiate women with FMS from those with other localized/regional chronic pain conditions. Depressive/anxiety levels and sleep quality exhibited ROC values > 0.9, meaning that they have good discriminatory power to differentiate between women with and without FMS; however, their specificity was lower than 20%, limiting their ability to exclude the presence of these symptoms in other chronic pain conditions. These results could be related to the fact that the PROMs used in the current study evaluating psychological and cognitive aspects are generic and not exclusive to FMS. Thus, pain hypervigilance, although it has the lowest ROC value (0.798) of all the PROMs used in our study, exhibited higher specificity (34.2%) than the remaining PROMs. These findings may be related to the presence of generalized hypervigilance as a perceptual maladaptive process of pain amplification in FMS patients [33]. In addition, higher levels of pain hypervigilance have been significantly associated with greater pain intensity and related disability [34]. In fact, our regression analyses also found that pain hypervigilance was a predictor of pain intensity within the FMS group and of disability within the non-FMS group. Accordingly, it is possible that pain hypervigilance represents a cognitive domain more associated with pain and disability in both widespread chronic pain conditions such as FMS and also localized/regional chronic pain conditions, since pain hypervigilance is an exclusive domain of FMS.

Based on the current results, the combination of the ACR diagnostic criteria and these PROMs may be able to better discriminate between women with FMS and those with other chronic pain conditions that could share some similarity with FMS. Studies investigating the relevance of including these variables in the ACR diagnostic criteria of FMS in clinical settings are needed.

### 4.3. Limitations

The results of the current study must be interpreted considering its limitations. First, we only included women with FMS, so the diagnostic accuracy of the PROMs investigated should not be applied to men with FMS. Second, we used generic PROMs for assessing psychological (HADS) and cognitive (PVAQ-9, PSQI)-related variables. These PROMs are also extensively used in other chronic pain conditions; hence, although their accuracy can be considered appropriate, they are not exclusive features of FMS. Thus, it should be noted that the FIQ is a disease-specific PROM; accordingly, its diagnostic accuracy could be biased towards those symptoms most reported by patients with FMS. Finally, the results of the current study must be considered according to clinical features of both cohorts. For instance, we do not know if a cohort of patients with localized/regional pain with higher levels of pain intensity would lead to similar results.

## 5. Conclusions

This study found that women with FMS exhibit worse clinical, psychological, and cognitive-related variables than women with localized/regional chronic pain. Additionally, all PROMs showed good discriminatory power, but related disability and pain hypervigilance were those variables showing the best performance. Nevertheless, the specificity values were extremely low, suggesting that although these variables can be useful for identifying the presence of FMS, they do not reliably exclude the possibility of its absence in women with other chronic pain conditions. It is possible that the combination of ACR diagnostic criteria with these PROMs can led to better discrimination between women with and without FMS.

## Figures and Tables

**Table 1:** Means and standard deviations (SDs) of clinical, psychological, and cognitive-related variables in women with and without fibromyalgia (FMS).

	FMS (*n* = 129)	No FMS (*n* = 65)	*p*
Age (years)	59.3 (11.0)	59.7 (9.5)	0.943
Height (cm)	162.5 (7.0)	161.5 (6.5)	0.239
Weight (kg)	72.7 (15.0)	67.0 (11.7)	0.008
Time with Pain (years)	14.7 (5.9)	10.0 (5.2)	<0.001
Pain Intensity (NPRS, 0–10)	7.6 (1.3)	3.1 (2.1)	<0.001
Disability (FIQ, 0–100)	75.2 (12.2)	22.7 (16.3)	<0.001
Anxiety (HADS-A, 0–21)	13.5 (3.9)	5.9 (4.1)	<0.001
Depression (HADS-D, 0–21)	11.0 (4.3)	3.5 (3.6)	<0.001
Sleep (PSQI, 0–21)	15.2 (3.7)	7.2 (4.1)	<0.001
Pain Hypervigilance (PVAQ-9, 0–45)	29.4 (7.8)	16.3 (12.5)	<0.001

NPRS: Numerical Pain Rating Scale; FIQ: Fibromyalgia Impact Questionnaire; HADS: Hospital Anxiety and Depression Scale (A: anxiety, D: depression); PSQI: Pittsburgh Sleep Quality Index; PVAQ-9: Pain Vigilance and Awareness Questionnaire.

**Table 2:** Discriminant capacity of clinical, psychological, and cognitive-related variables to differentiate women with and without fibromyalgia.

Variables	ROC Value	95% CI	Cut-Off Point	Significance	Sensitivity	Specificity	Youden Index	Positive LR	Negative LR
Pain Intensity (NPRS)	0.987	0.976–0.997	5.9	0.000	0.915	0.036	0.879	0.949	2.361
Related Disability (FIQ)	0.980	0.963–0.997	51.8	0.000	0.938	0.054	0.876	0.992	1.148
Anxiety Levels (HADS-A)	0.901	0.863–0.938	10.5	0.000	0.814	0.171	0.643	0.982	1.088
Depressive Levels (HADS-D)	0.906	0.868–0.944	6.5	0.000	0.853	0.180	0.673	1.040	0.817
Pain Hypervigilance (PVAQ-9)	0.798	0.740–0.856	22.5	0.000	0.806	0.342	0.464	1.225	0.567
Sleep Quality (PSQI)	0.909	0.872–0.946	12.5	0.000	0.791	0.117	0.674	0.896	1.786

NPRS: Numerical Pain Rating Scale; FIQ: Fibromyalgia Impact Questionnaire; HADS: Hospital Anxiety and Depression Scale (A: anxiety; D: depression); PVAQ-9: Pain Vigilance and Awareness Questionnaire; PSQI: Pittsburg Sleep Quality Index.

**Table 3:** Pearson product–moment correlation matrix between clinical, psychological, and cognitive-related variables in women with fibromyalgia.

	1	2	3	4	5	6
1. Years with Pain						
2. Pain Intensity (NPRS)	n.s.					
3. Disability (FIQ)	n.s.	0.456 **				
4. Anxiety (HADS-A)	n.s.	0.404 **	0.500 **			
5. Depression (HADS-D)	n.s.	0.339 **	0.512 **	0.588 **		
6. Pain Hypervigilance (PVAQ-9)	n.s.	0.420 **	0.276 **	0.393 **	0.326 **	
7. Sleep Quality (PSQI)	n.s.	0.378 **	0.482 **	0.602 **	0.555 **	0.389 **

NPRS: Numerical Pain Rating Scale; FIQ: Fibromyalgia Impact Questionnaire; HADS: Hospital Anxiety and Depression Scale (A: anxiety; D: depression); PSQI: Pittsburgh Sleep Quality Index; PVAQ-9: Pain Vigilance and Awareness Questionnaire ** *p* < 0.01. n.s. non-significant.

**Table 4:** Pearson product–moment correlation matrix between clinical, psychological, and cognitive-related variables in women with localized/regional chronic pain.

	1	2	3	4	5	6
1. Years with Pain						
2. Pain Intensity (NPRS)	n.s.					
3. Disability (FIQ)	n.s.	0.564 **				
4. Anxiety (HADS-A)	n.s.	0.457 **	0.745 **			
5. Depression (HADS-D)	n.s.	0.356 **	0.580 **	0.770 **		
6. Pain Hypervigilance (PVAQ-9)	n.s.	0.271 **	0.410 **	0.296 **	0.459 **	
7. Sleep Quality (PSQI)	n.s.	0.390 **	0.441 **	0.544 **	0.580 **	0.322 **

NPRS: Numerical Pain Rating Scale; FIQ: Fibromyalgia Impact Questionnaire; HADS: Hospital Anxiety and Depression Scale (A: anxiety; D: depression); PSQI: Pittsburgh Sleep Quality Index; PVAQ-9: Pain Vigilance and Awareness Questionnaire ** *p* < 0.01. n.s. non-significant.

**Table 5:** Summary of the stepwise regression analyses to determine predictors of pain intensity (NPRS) and disability (FIQ) in women with fibromyalgia syndrome.

	Predictor Outcome	Β	SE B	95% CI	*B*	t	*p*
Pain Intensity (NPRS)	Step 1						
	FIQ	0.049	0.008	0.032; 0.066	0.456	5.781	<0.001
	Step 2						
	FIQ	0.039	0.008	0.023; 0.056	0.367	4.724	<0.001
	PVAQ-9	0.053	0.013	0.027; 0.079	0.316	4.069	<0.001
	**Predictor Outcome**	**Β**	**SE B**	**95% CI**	***B***	**t**	***p***
Related Disability (FIQ)	Step 1						
	HADS-D	1.449	0.216	1.022; 1.876	0.512	6.718	<0.001
	Step 2						
	HADS-D	1.142	0.216	0.716; 1.569	0.404	5.298	<0.001
	Pain Intensity	2.976	0.710	1.571; 4.380	0.320	4.193	<0.001
	Step 3						
	HADS-D	0.860	0.243	0.379; 1.341	0.304	3.538	0.001
	Pain Intensity	2.566	0.719	1.141; 3.985	0.275	3.566	0.001
	PSQI	0.669	0.282	0.110; 1.228	0.207	2.370	0.019

NPRS: Numerical Pain Rating Scale; FIQ: Fibromyalgia Impact Questionnaire; HADS-D: Hospital Anxiety and Depression Scale for depression; PVAQ-9: Pain Vigilance and Awareness Questionnaire; PSQI: Pittsburg Sleep Quality Index. Pain Intensity Analysis: R^2^ adj. = 0.202 for step 1; R^2^ adj. = 0.289 for step 2. Related Disability Analysis: R^2^ adj. = 0.256 for step 1; R^2^ adj. = 0.342 for step 2; R^2^ adj. = 0.380 for step 3.

**Table 6:** Summary of stepwise regression analyses to determine predictors of pain intensity (NPRS) and disability (FIQ) in women with localized/regional pain.

	Predictor Outcome	Β	SE B	95% CI	*B*	t	*p*
Pain Intensity (NPRS)	Step 1						
	FIQ	0.067	0.012	0.042; 0.092	0.564	5.423	<0.001
	**Predictor Outcome**	**Β**	**SE B**	**95% CI**	***B***	**t**	***p***
Related Disability (FIQ)	Step 1						
	HADS-A	3.104	0.350	2.405; 3.802	0.745	8.875	<0.001
	Step 2						
	HADS-A	2.566	0.367	1.832; 3.300	0.616	6.987	<0.001
	Pain Intensity	2.381	0.744	0.894; 3.868	0.282	3.200	0.002
	Step 3						
	HADS-D	2.412	0.365	1.683; 3.142	0.579	6.611	<0.001
	Pain Intensity	2.134	0.734	0.667; 3.601	0.253	2.908	0.005
	PVAQ-9	0.256	0.122	0.013; 0.500	0.170	2.103	0.040

NPRS: Numerical Pain Rating Scale; FIQ: Fibromyalgia Impact Questionnaire; HADS-D: Hospital Anxiety and Depression Scale for depression; PVAQ-9: Pain Vigilance and Awareness Questionnaire. Pain Intensity Analysis: R^2^ adj. = 0.307 for step 1. Related Disability Analysis: R^2^ adj. = 0.549 for step 1; R^2^ adj. = 0.606 for step 2; R^2^ adj. = 0.627 for step 3.

## Data Availability

The materials and analysis code for this study are not available in any repository; however, we will make our data accessible upon request to the corresponding author.
